# Using Satellite Images of Environmental Changes to Predict Infectious Disease Outbreaks

**DOI:** 10.3201/eid/1509.081334

**Published:** 2009-09

**Authors:** Timothy E. Ford, Rita R. Colwell, Joan B. Rose, Stephen S. Morse, David J. Rogers, Terry L. Yates

**Affiliations:** University of New England, Biddeford, Maine, USA (T.E. Ford); University of Maryland, College Park, Maryland, USA (R.R. Colwell); Johns Hopkins University Bloomberg School of Public Health, Baltimore, Maryland, USA (R.R. Colwell); Michigan State University, East Lansing, Michigan, USA (J.B. Rose); Columbia University Mailman School of Public Health, New York, New York, USA (S.S. Morse); Oxford University, Oxford, UK (D.J. Rogers); University of New Mexico, Albuquerque, New Mexico, USA (T.L. Yates); 1 Deceased.

**Keywords:** Satellite imaging, cholera, prediction, global change, environment, hantavirus, malaria, parasites, modeling, perspective

## Abstract

Recent events clearly illustrate a continued vulnerability of large populations to infectious diseases, which is related to our changing human-constructed and natural environments. A single person with multidrug-resistant tuberculosis in 2007 provided a wake-up call to the United States and global public health infrastructure, as the health professionals and the public realized that today’s ease of airline travel can potentially expose hundreds of persons to an untreatable disease associated with an infectious agent. Ease of travel, population increase, population displacement, pollution, agricultural activity, changing socioeconomic structures, and international conflicts worldwide have each contributed to infectious disease events. Today, however, nothing is larger in scale, has more potential for long-term effects, and is more uncertain than the effects of climate change on infectious disease outbreaks, epidemics, and pandemics. We discuss advances in our ability to predict these events and, in particular, the critical role that satellite imaging could play in mounting an effective response.

## Abstract 2

A strong global satellite imaging system is essential for predicting outbreaks.

Atmospheric chemists and climate modelers have little doubt that the earth’s climate is changing. Concomitant with rising carbon dioxide levels and temperatures, severe weather events are increasing, which can lead to substantial rises in sea level, flooding, increased droughts, and forest fires (*1*). In recent decades, infectious diseases have resurged, and previously unrecognized agents of disease have been characterized (*2*). Evidence is accruing that these phenomena may in part be linked to environmental change (*3*). Several questions have emerged from events that have occurred over the past 20 years: was cryptosporidiosis inevitable in Milwaukee, Wisconsin, USA, in 1993, and was *Escherichia coli* O157 infection inevitable in Walkerton, Ontario, Canada, in 2000? Both events were preceded by heavy rains; had highly concentrated sources of pathogens in the form of untreated sewage and animal waste, respectively; and had vulnerable infrastructure. Although the situations were perhaps more complex, could we have predicted epidemic cholera in South America in 1991 after a 100-year absence and the emergence of a new strain of potentially pandemic cholera in India in 1992?

A considerable body of knowledge has accumulated over the past decade or so about the relationships between environment and disease, yet far more information and resources are needed if we are to develop effective early warning systems through environmental surveillance and modeling as well as appropriate emergency response. In the United States, we face a crisis in funding that not only affects basic and applied research in this field but also undermines our ability to deploy remote sensing technologies that provide the most promising means for monitoring our environment. Using examples of waterborne and vector-borne disease, we will discuss how remote sensing technology can be used for disease prediction. We will then examine the lessons learned from these examples and provide recommendations for future modeling.

## Waterborne Disease

Water and climate go hand in hand, with precipitation and extreme events known to be associated with waterborne outbreaks (*4*). Flooding is the most frequent natural weather disaster (30%–46% of natural disasters in 2004–2005), affecting >70 million persons worldwide each year (data for 2005 [*5*]).

The most common illnesses associated with floods described in the literature are diarrhea, cholera, typhoid, hepatitis (jaundice), and leptospirosis. Unusual illnesses such as tetanus have also been reported. The etiologic agents identified include *Cryptosporidium* spp., hepatitis A virus, hepatitis E virus, *Leptospira* spp., *Salmonella* spp*.*, and *Vibrio* spp. Severe outbreaks of cholera, in particular, have been directly associated with flooding in Africa and in West Bengal, India (*6*,*7*).

A rise in sea level, combined with increasingly severe weather events, is likely to make flooding events commonplace worldwide. The Climate Change 2001 Synthesis Report from the Intergovernmental Panel on Climate Change (*8*) suggests that the average annual numbers of persons affected by coastal storm surges will increase from <50 million at present sea levels to ≈250 million by the 2080s, assuming a 40-cm rise in sea level. Even with enhanced protection through engineering interventions, this number is anticipated to reach ≈100 million persons. The initial proportion of deaths from these events is huge, but without extreme vigilance and better monitoring and response, major epidemic waterborne diseases will continue to occur. Factors that promote waterborne disease—overcrowding, lack of sanitation, lack of clean water, certain domestic animal practices, waste disposal—are exacerbated by flooding.

## Using Satellite Technology to Model Prediction of Cholera Outbreaks

**Figure 1:**
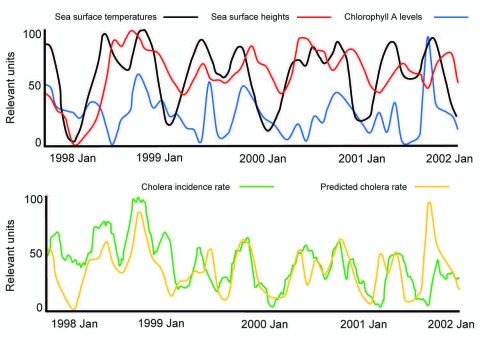
Modeling cholera outbreaks in Bangladesh. Adapted from R.R. Colwell and J. Calkins, unpub. data.

Effective prediction depends on many factors, not just the prediction of an event. Cholera may be the most studied and best understood of the waterborne diseases and, perhaps in hindsight, we could have predicted the occurrence of cholera in South America in 1991 (*9*). Models for cholera prediction, although country specific, are constantly improving. For example, considerable work has gone into predicting outbreaks of cholera in Bangladesh. Remote imaging technologies developed by the US National Aeronautics and Space Administration have been used to relate sea surface temperature, sea surface height, and chlorophyll A levels to cholera outbreaks (Figure 1) (R.R. Colwell and J. Calkins, unpub. data). This process used a composite environmental model that demonstrated a remarkable similarity between predicted rates based on these 3 parameters and actual cholera incidence. These data are far from perfect and considerable uncertainty still remains. For example, rates of cholera were much higher than predicted in January 1998 and January 1999, yet many of the predicted peaks closely aligned with actual incidence. Because the model is constantly being improved and the satellite data are becoming increasingly accurate through ground truthing (real-time collection of information on location), we believe that satellite imaging provides tremendous promise for prediction of cholera, weeks and even months in advance of an epidemic.

Knowing when an outbreak is likely to occur can inform public health workers to stress basic hygiene and sanitation and to implement simple mitigation efforts such as filtration of water with sari cloth, which in some areas is credited with reducing deaths from cholera by >50% (*10*). Although remote sensing technology is currently still a research tool, the example of cholera prediction through its use provides a compelling argument to maintain and adequately fund our satellite programs; unless this is done, this extraordinary effort at disease prediction will fail.

Some of the critical needs that must be met to predict the effect of environmental change on waterborne disease include the following: 1) better knowledge of disease incidence and pathogen excretion; 2) better characterization of the pathogens in sources (e.g., combined sewer overflows, septic tanks) and these sources’ vulnerabilities to climate change; 3) better monitoring of sewage indicators to gather source, transport, and exposure information (event monitoring); 4) improved understanding of sediments and other pathogen reservoirs; 5) more quantitative data for risk assessment; and 6) better health surveillance data. In turn, this information can be used to better use ground truthing in combination with remote sensing technologies as predictors of waterborne disease outbreaks.

## Vector-borne Disease

Other emerging and reemerging infectious diseases also are environmentally driven. Many are zoonotic, vector-borne, or both, and have complex life histories that make predicting disease emergence or reemergence particularly difficult. An insect or rodent vector can make it almost inevitable that a pathogen will be globally transported by plane or boat. With environmental change, disease range, prevalence, and seasonality may change in direct relationship to the vector or animal host. Therefore, to understand the life cycle of a pathogen and the risks of disease emergence, all stages of that life cycle and the life cycles of its intermediate hosts must be considered.

To date, predicting vector-borne diseases has proved to be complex. Although climate change and other environmental stressors are major components, separation from human factors is difficult. Climate change undoubtedly affects the distribution of disease, but changes in human behavior that increase exposure risk are also critical factors. Šumilo et al. (*11*) reported that climatic variables explain only 55% of spatial variation in tick-borne encephalitis in the Baltic States, which have seen an increase in disease incidence over the past 3 decades. These authors report that changes in predation pressure on intermediate hosts and shifting socioeconomic conditions that increase or decrease peoples’ visits to forests (for recreation, work, or berry and mushroom harvesting) are important factors in disease distribution (*12*).

Effective modeling of future risk for vector-borne disease outbreaks needs to take into account human behavior that increases exposure, as well as other factors that effect the ecology of the vectors, such as predation pressure and habitat change. Coupled with remote sensing technologies that monitor environmental and climatic changes, human observations of population movement and distribution will be necessary.

Malaria also presents a challenge. This disease continues to devastate sub-Saharan Africa and other parts of the developing world. Substantial resources over the past several decades have gone toward eradication, vaccination, treatment, and, more recently, prediction of malaria outbreaks. Satellite imaging has been used to predict the distribution of 5 of the 6 *Anopheles gambiae* complex species that are responsible for much of the malaria transmission in Africa (*13*). However, human factors again make accurate prediction of disease events complex. Prediction of a disease event is complicated by host immunity effects, which can result in cycles of infection that would appear to bear no relationship to environmental variables. To predict malaria outbreaks, remote sensing technologies need to be coupled with a better understanding of how specific populations are effected by host immunity, which could allow population susceptibility at any given time to be estimated.

## Using Satellite Technology to Model Hantavirus Pulmonary Syndrome

Although considerable uncertainty exists in disease prediction through remote sensing technology, particularly for vector-borne disease as discussed above, satellite technology has been applied with some success to predictive modeling for cases of hantavirus pulmonary syndrome (HPS). The 1993 outbreak of HPS in the southwestern United States was believed to be linked to environmental conditions and, in particular, to abnormally high rainfall that resulted in increased vegetation with a subsequent explosion in the rodent populations. Several research groups have subsequently modeled conditions that led to an HPS outbreak, with mixed success. Engelthaler et al. (*14*) looked at 10 years of data on monthly precipitation and daily ambient temperature in the Southwest region (1986–1995) in relation to HPS cases (1993–1995). They found that cases tended to cluster seasonally and temporally by biome type and elevation and only indirectly demonstrated a possible association between the 1992/1993 El Niño precipitation events and HPS. Glass et al. (*15*,*16*) were also unable to make a definitive link with precipitation events in their analyses of HPS in the southwestern United States. They did, however, find a relationship between Landsat Thermatic Mapper (LTM) images recorded by satellite in 1992 and HPS risk the following year. LTM generates numbers that represent reflected light in 6 bands, 2 of which were associated with decreased risk and 1, in the mid-infrared range, with increased risk. The authors admit that considerable ground truthing is necessary to relate satellite imagery to the environmental variables being measured (i.e., vegetation, soil type, soil moisture) and their relation to rodent population dynamics.

However, this work does demonstrate the utility of remote satellite imaging and the increasingly important role it can and should play in disease prediction. In 2006, Glass et al. (*17*) reported strong predictive strength from logistic regression modeling of LTM imagery from 1 year, when estimating risk of HPS the following year, for the years 1992–2005. Their risk analysis for 2006, based on Landsat imagery for 2005, when precipitation levels increased dramatically over prior drought years, suggested an increased risk for HPS, particularly in northern New Mexico and southern Colorado. This prediction was unfortunately borne out in the early part of 2006 when 9 cases of HPS occurred within the first 3 months, 6 of those cases in New Mexico and Arizona. However, the anticipated threat to Colorado did not occur, with a fairly typical number of 6 cases, compared with a total of 11 cases for the state in 2005 (*18*).

However, these results are not necessarily a failure of prediction. In fact, they may illustrate that an early warning system serves to reduce exposure of persons to the deer mice habitat. For example, USA Today highlighted HPS risks with a June 8, 2006, article titled “Officials warn of increased threat of hantavirus” (www.usatoday.com/news/health/2006-06-08-hantavirus-x.htm). The role of the popular press is hard to quantify but undoubtedly does have an effect on human behavior patterns. Many health departments in the western states produce health advisories warning the public about the risks of exposure to the virus through inhalation of dust contaminated with rodent urine, feces, or saliva. The popular press may serve an important role in increasing awareness of a heightened health risk, which, in turn, promotes greater compliance with health advisories.

## Lessons Learned and Recommendations for Future Modeling

The scientific community has a relative consensus that epidemic and pandemic disease risks will be exacerbated by environmental changes that destabilize weather patterns, change distribution of vectors, and increase transport and transmission risk. Predictive modeling may lead to improved understanding and potentially prevent future epidemic and pandemic disease. Many respiratory infections are well known as highly climate dependent or seasonal. Although we are not yet able to predict their incidence with great precision, we may well be able to do this in the future. Meningococcal meningitis (caused by *Neisseria meningitidis*) in Africa is probably the best known example. In the disease-endemic so-called meningitis belt (an area running across sub-Saharan Africa from Senegal to Ethiopia), this is classically a dry season disease, which ceases with the beginning of the rainy season, likely as a result of changes in host susceptibility (*19*). Many other infectious diseases show strong seasonality or association with climatic conditions (*20*). Perhaps one of the most interesting is influenza, which is thought of as a wintertime disease in temperate climates but shows both winter and summer peaks in subtropical and tropical regions (*21*). Although the reasons for seasonality are often poorly understood, the close dependence of such diseases on climatic conditions suggests that these, too, are likely to be amenable to prediction by modeling and remote sensing (*22*).

When we consider influenza, it is hard not to think about the future risks from pandemic influenza. Public health agencies in the United States and around the world are focusing on influenza preparedness, notably concerning influenza virus A subtype H5N1, which has captured attention because it causes severe disease and death in humans but as yet has demonstrated only very limited and inefficient human-to-human transmission. The severity of the disease raises images of the 1918 influenza epidemic on an unimaginably vast scale if the virus were to adapt to more efficient human-to-human transmission. Can predictive modeling using satellite or other imaging of environmental variables help in prediction of future influenza pandemics? Xiangming Xiao at the University of New Hampshire was funded in 2006 by the National Institutes for Health to lead a multidisciplinary and multi-institutional team to use remote satellite imaging to track avian flu. Xiao et al. have used satellite image–derived vegetation indices to map paddy rice agriculture in southern Asia (*23*). They believe that a similar approach can be used in conjunction with the more traditional approach of analyzing bird migration patterns and poultry production (*24*,*25*) to map potential hot spots of virus transmission (*26*).

An interesting question is why did we not see disease epidemics in Indonesia, following the devastating tsunami disaster of December 2004? Could rapid public health intervention be credited with minimizing spread of disease? In the case of Aceh Province, many communities reported diarrhea as the main cause of illness (in 85% of children <5 years of age), but no increases in deaths were reported, and no outbreaks of cholera or other potentially epidemic diseases occurred (*27*). Given the massive scale of the disaster, was this likely? In some towns, more than two thirds of the population died at the time of impact, almost 100% of homes were destroyed, and 100% of the population lacked access to clean water and sanitation (*27*). To a large extent, the Australian army and other groups are to be credited with rapidly deploying environmental health teams to swiftly implement public health measures, including provision of safe drinking water, proper sanitary facilities, and mosquito control measures (*28*). Widespread fecal pollution of the surface waters was shown, yet the saltiness of the potable water supply after the disaster made much of the water unpalatable. Wells were vulnerable, perhaps to other etiologic agents of fecal origin including viruses and *Shigella* spp*.*, with greater probability of infection than *Vibrio* spp., thus leading to the widespread diarrhea.

The most important lesson from the Asian tsunami is that disease epidemics can be prevented by public health intervention. Unfortunately, most flooding events, and other conditions that promote infectious disease epidemics, do not receive the same global media attention. A tsunami captures the imagination of the world in a way that weeks of rainfall in the Sudan or a rise in sea surface temperature cannot. However, if climatologic data can be used to predict future disease outbreaks, public health interventions can be mobilized in a more timely and proactive manner.

A continuing concern is the conditions that result in newly emergent virulent strains of pathogens. Faruque et al. have provided molecular evidence that *V. cholerae* O139 strains are derived from O1 strains through genetic modification (*29*). In addition, Chakraborty et al. in Kolkata have seen the presence and expression of virulence genes in several environmental strains of *V. cholerae* cultured from surface waters (*30*). Recently, *E. coli* O157 has been isolated from the Ganges River in India for the first time (*31*). Indications are that it is metabolically different from *E. coli* O157 isolated from other parts of the world, but the conditions that have led to these differences are as yet unclear. From the above studies, risk for transmission of virulence genes is likely to be high, but studies of conditions promoting transmission and approaches to modeling resultant disease risks are in their infancy. New epidemic strains could potentially occur through mutation of existing epidemic strains or through gene transfer. Environmental stressors such as chemical contaminants are thought to accelerate both mutation rates and gene transfer (*32*). Thus, the degree of chemical pollution may need to be a component of disease models (in addition to other stressors).

The scientific community is a long way from incorporating environment-gene interactions into predictive models and clarifying the risks posed to human society from emerging diseases. However, investigation of these parts of the pathogen’s ecology should remain on the national research agenda as we move forward with developing predictive models of disease outbreaks.

**Figure 2:**
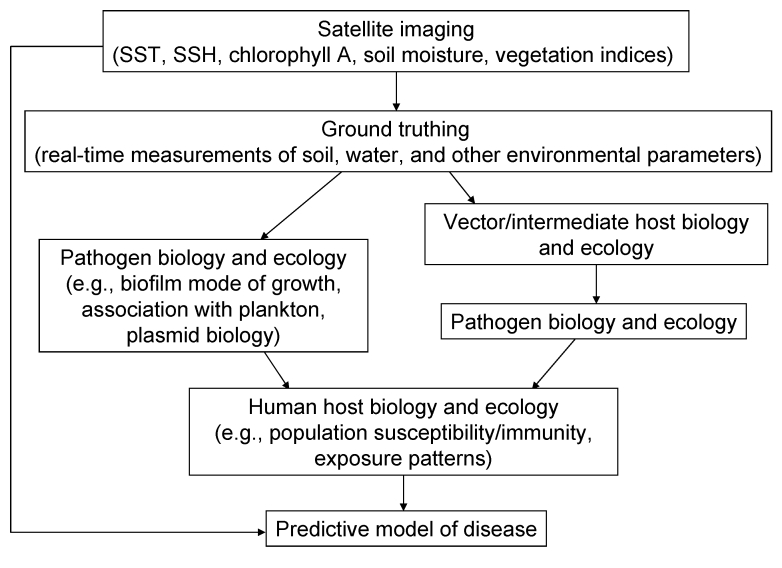
Components of a predictive model of infectious disease based on satellite imaging to assess environmental change. SST, sea surface temperature; SSH, sea surface height.

Current modeling of infectious diseases is by necessity retrospective. Environmental parameters measured by remote satellite imaging show the greatest promise for providing global coverage of changing environmental conditions. With current imaging technologies, we can measure sea surface temperature, sea surface height, chlorophyll A levels, and a variety of vegetation and soil indices, in addition to many other physical, biologic, and chemical parameters of the earth’s surface and atmosphere. A variety of these parameters can be incorporated in complex mathematical models, together with biotic and ecologic variables of the pathogen and host life cycles, to correlate environment with outbreaks of disease (Figure 2). However, we are still far from being able to accurately predict future disease events on the basis of existing environmental conditions.

Successful predictive modeling of disease and the establishment of early warning systems have reached a critical junction in development. As we improve our understanding of the biology and ecology of the pathogen, vectors, and hosts, our ability to accurately link environmental variables, particularly those related to climate change, will improve. What has become clear over the past few years is that satellite imaging can play a critical role in disease prediction and, therefore, inform our response to future outbreaks.

We conclude that infectious disease events may be closely linked to environmental and global change. Satellite imaging may be critical for effective disease prediction and thus future mitigation of epidemic and pandemic diseases. We cannot stress too strongly our belief that a strong global satellite program is essential for future disease prediction.
